# Botulinum Toxin for the Treatment of Motor Tics in Children: A Case Series

**DOI:** 10.1177/08830738251360210

**Published:** 2025-08-06

**Authors:** Ethan Edmondson, Mariam Hull, Sukru Aras, Mered Parnes

**Affiliations:** 1 Pediatric Movement Disorders Clinic, Section of Pediatric Neurology and Developmental Neuroscience, 3984 Texas Children's Hospital and Baylor College of Medicine, Houston, TX, USA; 2 Department of Pediatrics–Neurology Bioinformatics, 3989 Baylor College of Medicine, Houston, TX, USA

**Keywords:** pediatric, quality of life, Tourette syndrome, treatment

## Abstract

Tic disorders, including Tourette syndrome, are characterized by brief, repetitive, and patterned movements or vocalizations. Botulinum neurotoxin (BoNT) has emerged as a therapeutic option for motor tics, particularly when oral medications are insufficient or poorly tolerated. This retrospective cohort study evaluated the safety and effectiveness of botulinum neurotoxin injections in 50 pediatric patients (aged 7-18 years) with motor tic disorders treated at a tertiary pediatric movement disorders clinic between May 2019 and January 2024. Botulinum neurotoxin injections were tailored to individual symptomatology. Overall, 64% of patients reported improvement in tic severity, 36% noted no change, and 6% worsened. Response did not differ significantly by tic complexity, phenomenology (clonic vs dystonic), or presence of a premonitory urge. Comorbidities such as attention-deficit hyperactivity disorder (ADHD), obsessive-compulsive disorder, autism, and intellectual disability were not predictive of outcomes. These findings support botulinum neurotoxin as a safe and potentially effective treatment for pediatric motor tics, including complex and malignant presentations.

Tic disorders are defined by sudden, brief, repetitive, and stereotyped movements or vocalizations. Tourette syndrome (TS), a neurobehavioral condition characterized by both motor and vocal tics, with onset of tics in childhood, affects an estimated 0.3% to 0.9% of children.^1,2^ Males are disproportionately affected, with a male-to-female ratio of 3:1.^3^ Tic disorders have a strong genetic basis, with the risk of developing these conditions increasing in proportion to the genetic proximity of relatives.^4^ Tics frequently co-occur with neuropsychiatric comorbidities such as attention-deficit hyperactivity disorder (ADHD) and obsessive-compulsive disorder. Tics tend to wax and wane, although for many they peak in intensity and frequency near age 12 years.^5-9^

The clinical presentation of tics varies widely, ranging from mild and nondisruptive to severe and debilitating. A simple tic is a sudden, brief, repetitive movement or sound involving only a few muscle groups, like blinking or sniffing, whereas a complex tic is a coordinated pattern of movements or phrases. Motor tic phenomenology varies as well between clonic tics—sudden, jerklike movements—and dystonic tics—slower, sustained, twisting, or posturing motor tics that may appear more forceful or prolonged. Although for some patients, tics are not bothersome or impairing and require no treatment, for others, tics may interfere with participation in activities, schoolwork, or other daily tasks. Repetitive motor tics can cause pain, and vocalizations can interfere with speech and call unwanted attention.^10-12^ Additionally, tics can produce social and psychological consequences, such as stigmatization, isolation, and diminished quality of life. These challenges underscore the need for effective, accessible treatment strategies that address both the physical and psychosocial dimensions of tic disorders.^13-16^ For some, tics can be so severe that they cause significant self-injury and morbidity, referred to as “malignant tics.” Several cases have been documented in the literature describing forceful and repetitive “whiplash” flexion and extension movements of the neck that caused a progressive cervical myelopathy and resultant impairments in ambulation and mobility.^17,18^

A hallmark feature of tics is the premonitory urge, an uncomfortable sensory or mental phenomenon experienced by up to 90% of patients.^19,20^ These urges are typically described as regional or generalized sensations that precede the execution of a tic and are temporarily relieved by performing it.^21-23^ The urge-tic cycle is integral to the disorder's symptomatology, as patients often perceive their tics as voluntary responses to involuntary sensations. This phenomenon is especially pronounced in individuals with comorbid obsessive-compulsive disorder, where motor tics may be performed repeatedly to achieve relief that feels “just right.”^24^

Over the years, the treatment of tic disorders has evolved significantly, reflecting advances in both pharmacologic and nonpharmacologic interventions. In the early 20th century, neuroleptics such as haloperidol and pimozide were among the first medications used to control tics, based on their dopamine-blocking properties.^25^ These treatments, although effective in reducing tic severity, can be associated with side effects such as sedation, weight gain, drug-induced parkinsonism, and tardive dyskinesia, which led to a demand for alternative therapies.

The modern treatment landscape for tics includes behavioral interventions, pharmacologic therapies, and, in severe and malignant cases, surgical approaches. Comprehensive behavioral intervention for tics remains the mainstay of nonpharmacologic treatment, whereas medications such as alpha-2-agonists (eg, guanfacine, clonidine), dopamine receptor antagonists (eg, fluphenazine, aripiprazole), and VMAT2 inhibitors (eg, tetrabenazine, valbenazine) are commonly used.^26,27^ However, these treatments are not universally effective, and many patients face barriers such as limited access to trained behavioral therapists, medication side effects, and contraindications to surgery. Although deep brain stimulation (DBS) surgery for tics can be effective, the implantation of permanent intracranial hardware is not appropriate for all pediatric patients and is complicated by the tendency for tics to plateau and improve in early adulthood.

In this context, botulinum neurotoxin has emerged as a promising adjunct or alternative therapy for tic disorders.^28-30^ Botulinum neurotoxin, widely used in the management of movement disorders such as dystonia and spasticity, functions by inhibiting acetylcholine release at the neuromuscular junction, leading to targeted muscle relaxation. Early studies and clinical experience suggest that botulinum neurotoxin not only reduces tic frequency and intensity but also alleviates premonitory urges, offering relief for patients whose tics are refractory to other treatments.^31,32^

The first use of botulinum neurotoxin for the treatment of tics was reported in 1991, in which a patient with a 15-year history of a bothersome complex tic was described, composed of contraction of the frontalis and scalp followed by a nod. The frontalis muscle was injected to lessen the force of the movement. The author may have been surprised to learn that not only was the frontalis and scalp movement eliminated, but the urge was as well, as was the nod. The complex tic did not return after a year of follow-up.^33,34^ The first case series followed in 1994, focusing on the use of botulinum toxin for treating dystonic tics, given its known efficacy in managing focal dystonia.^35^ This study involved 10 male patients, aged between 13 and 53 years, each presenting with severe focal tics—5 with dystonic eye closure and 5 with painful dystonic neck tics. Botulinum neurotoxin injections were administered to the pretarsal orbicularis oculi and the cervical muscles exhibiting the most palpable contractions, with consideration given to the locations of premonitory sensations. Doses comparable to those used for dystonia were used, and patients were evaluated using a 0 to 4 clinical rating scale. Each patient received between 1 and 5 injection sessions, totaling 29 sets of injections across the cohort. All 10 patients experienced moderate or greater improvement in the frequency and intensity of their tics, with benefits lasting between 2 and 20 weeks. Transient adverse effects occurred following 7 of the 29 injection sessions, including ptosis, neck pain or stiffness, and neck weakness; however, these side effects resolved within weeks, and no serious complications were reported. Also noted was an unexpected improvement of the premonitory urge. It was theorized that peripheral sensory mechanisms play a role in the pathophysiology of tics, which perhaps were somehow interrupted by botulinum neurotoxin.

In a series of 35 patients with persistent tics (30 male), 34 met criteria for Tourette syndrome, age 23.3 ± 15.5 years (range, 8-69 years) who were injected over 115 visits, the mean peak effect was 2.8 (range, 0-4), mean duration of benefit was 14.4 weeks (maximum, 45 weeks), and mean latency to onset of benefit was 3.8 days (maximum, 10 days). Twenty-one (84%) of 25 patients with premonitory sensations derived marked relief of those symptoms (mean benefit 70.6%). Complications included neck weakness (4/35), dysphagia (2), ptosis (2), nausea (1), hypophonia (1), fatigue (1), and generalized weakness (1), which were all mild and transient.^36^

Most studies to date have focused on adults, leaving gaps in understanding the efficacy, safety, and optimal use of this treatment for children with tics. Here we present a series of pediatric patients treated with botulinum neurotoxin for motor tics. Numerous studies have also demonstrated botulinum neurotoxin injections into the vocal cords could reduce tic frequency in patients with refractory phonic tics, including complex, socially harmful vocalizations such as malignant coprolalia. Given that our otolaryngology colleagues manage vocal cord injections at our institution, we have limited our analysis to those patients treated for motor tics. By evaluating both efficacy and safety, this research aims to inform clinical decision making and expand treatment options for children with tic disorders.

## Materials and Methods

This study is a retrospective cohort study of pediatric patients with motor tic disorders treated with onabotulinumtoxinA (BoNT-A; Botox) between May 2019 and January 2024 at a tertiary pediatric movement disorders clinic. Patients were included if they were <18 years of age at the time of treatment, had a confirmed clinical diagnosis of a tic disorder (Tourette syndrome, chronic motor tic disorder, or tics of presumed organic etiology), and received at least 1 botulinum neurotoxin injection specifically targeting motor tics. Patients with isolated phonic tics or who did not receive botulinum neurotoxin injections were excluded.

The primary outcome was caregiver- or patient-reported change in tic severity at 3-month follow-up, assessed using a 3-point ordinal scale: −1 (worsening), 0 (no change), and 1 (improvement). Secondary outcomes included qualitative caregiver reports on functional impact and adverse effects. Data were abstracted from clinical documentation in the electronic medical record, including demographics, comorbidities, tic phenomenology, treatment notes, and follow-up assessments.

Botulinum neurotoxin injections were administered following a comprehensive clinical evaluation by pediatric neurologists with fellowship training in movement disorders. Decisions to initiate treatment were made collaboratively with families through a shared decision-making process. Treatment was reserved for patients whose tics were persistent and functionally impairing. Families were routinely counseled that botulinum neurotoxin is not curative, and the goal of treatment is symptomatic reduction of bothersome or harmful tics.

Injection protocols were individualized based on tic phenomenology, distribution, and muscle involvement, consistent with established clinical practice and prior reports by Lang and Jankovic.^37^ Target muscle selection was guided by direct clinical observation and caregiver/patient history, including the presence of premonitory urges. Dosages were titrated based on the size and force of the involved muscle group, with a maximum cumulative dose not exceeding 10 units per kilogram of body weight.

Follow-up visits were scheduled at regular intervals with repeat injections no sooner than 12 weeks. At each follow-up, caregivers and/or patients were asked to report perceived benefits and adverse effects, including muscle weakness, voice changes, or injection site pain.

Statistical analysis was conducted to explore associations between clinical and demographic factors (age, sex, comorbid ADHD, autism, presence of premonitory urge, and dystonic vs clonic tic phenomenology) and treatment outcomes. Categorical variables were summarized using frequencies and proportions; continuous variables were reported as medians with interquartile ranges (IQRs). Fisher exact test and Kruskal-Wallis test were used to evaluate group differences. Analyses were performed using Stata version 18.5 (StataCorp LLC, College Station, TX) and SPSS version 29 (IBM Corp, Armonk, NY). A *P* value of <.05 was considered statistically significant (Table 1).

**Table 1.** Baseline Characteristics of Patients Treated With Botulinum Neurotoxin for Motor Tics.

Characteristics	All Subjects, n (%) (n = 50)
Gender	
Male	37 (74)
Female	13 (26)
Diagnosis	
TS	27 (54)
TSND	12 (24)
CMTD	11 (22)
Comorbidities	
ADHD	33 (66)
OCD	11 (22)
ID	15 (30)
Autism	8 (16)

Abbreviations: ADHD, attention-deficit hyperactivity disorder; CMTD, chronic motor tic disorder; OCD, obsessive-compulsive disorder; ID, intellectual disability; TS, Tourette syndrome; TSND, tic disorder secondary to another neurologic diagnosis.

## Results

This retrospective cohort study included all 50 pediatric patients, aged 7-18 years (mean age 12.8 years, SD ± 2.49), diagnosed with motor tic disorders who received botulinum neurotoxin (botulinum neurotoxin) injections as part of their clinical management at a pediatric movement disorders clinic between May 19, 2019, and January 1, 2024. The median age at first injection was 12.6 years (IQR: 10.8-14.5). Nearly two-thirds (64%) of patients received their first injection between the ages of 10 and 14 years, a developmental window during which tics often peak and may begin to remit as part of the natural history of Tourette syndrome. The median time between the clinical decision to inject with botulinum neurotoxin and intervention, as a result of scheduling logistics and insurance authorization processes, was 4 weeks. Clinic patients who did not undergo botulinum neurotoxin injections were excluded. botulinum neurotoxin injections for phonic tics are performed by an otolaryngologist rather than in movement disorders clinic, and therefore phonic tics were excluded from this research. The cohort consisted of 37 males (74%) and 13 females (26%). Twenty-seven patients (54%) met diagnostic criteria for Tourette syndrome, 12 (24%) had tic disorder secondary to another neurologic diagnosis, and 11 (22%) had a chronic motor tic disorder. Comorbidities were common, including ADHD in 33 patients (66%), intellectual disability in 15 (30%), obsessive-compulsive disorder in 11 (22%), and autism in 8 (16%). The average total botulinum neurotoxin dose administered per patient was 217 units (SD ± 137), with doses individualized by site and muscle group. Table 2 provides a detailed breakdown of the injection sites and average doses per region, ranging from facial muscles to paraspinals and lower extremity musculature.

**Table 2.** Summary of Botulinum Neurotoxin Doses Administered.

Body Region	Site Injected	Average Dose and Range per Site (U)
Face	Frontalis	10
	Procerus	10
	Corrugator	12.3 [10-15]
	Upper eyelid	11 [10-12]
	Lateral canthus	8
	Lower eyelid	11 [10-12
	Nasalis	8
	Masseter	50
	Submental	47.5 [25-70]
Neck	Splenius capitis	66.7 [50-75]
	Scalene	42 [25-75]
	Sternocleidomastoid	46 [30-75]
Trunk	Trapezius	90 [50-125]
	Thoracic paraspinals	150
	Lumbar paraspinals	100
Upper extremity	Triceps	45
	Supinator	40
	Flexor carpi radialis	50
	Flexor carpi ulnaris	50
Lower extremity	Gluteus medius and gluteus minimus	100
	Extensor hallucis longus	80
	Flexor hallucis longus and brevis	60

Thirty-two patients (64%) experienced improvement in the severity of motor tics treated by botulinum neurotoxin injections, 18 (36%) reported no change, and 3 patients (6%) noted worsening symptoms. Only two patients (4%) reported injection site weakness. One of those 2 patients reported dysphagia at follow-up 4 weeks after injection of 50 units to the sternocleidomastoid muscles bilaterally and 50 units to the submental complex. Videofluoroscopic swallow study was negative for this patient, and dysphagia had resolved at follow-up 3 months after these injections. No severe or systemic side effects were reported, supporting the favorable safety profile of botulinum neurotoxin in this pediatric population. Twenty-six of the 32 patients who improved have returned for follow-up injections by the closing date of our retrospective study period (81%). This subset of patients returned for an average of 3 follow-up botulinum neurotoxin procedure visits. Only 3 of the 15 patients whose condition had neither improved nor worsened were injected after the initial procedure date (20%). None of the 3 patients who reported worsening symptoms after the initial procedure were injected again within the study period.

Both dystonic and clonic tics responded to botulinum neurotoxin with similar efficacy. Among the 24 patients with dystonic tics, 54% reported symptom improvement, whereas 73% of the 26 patients with clonic tics noted similar benefits. This difference was not statistically significant (*P* = .16). Of the total cohort, 11 patients (22%) received botulinum neurotoxin for complex tics—defined as coordinated movements involving multiple muscle groups—whereas 39 (78%) were treated for simple tics. Outcomes between these 2 groups were comparable, with no meaningful difference in treatment response. The presence of a premonitory urge did not appear to predict response; among the 32 patients who reported a premonitory urge, 62.5% improved, compared with 66.7% of the 18 patients who did not report one. These findings should be interpreted cautiously, as reduced awareness or limited insight—particularly in younger or neurodivergent children—may account for underreporting of sensory phenomena in this population.

Eight patients in the cohort had forceful, malignant tics involving rapid flexion or extension of the neck. Six of these patients improved following treatment, whereas one reported no benefit and another, who had comorbid obsessive-compulsive disorder, experienced worsening symptoms. The latter case involved an inability to satisfy the compulsion to perform the tic “just right,” which led to distress following injection. Additional variables—including age at the time of injection, presence of intellectual disability, autism, or obsessive-compulsive disorder, and use of adjunct therapies—did not significantly influence treatment outcomes. Of the 50 patients, only 6 were not receiving oral medications including alpha-2 adrenergic agonists (guanfacine and clonidine), topiramate, dopamine blocking medications (pimozide, risperidone, aripiprazole, and fluphenazine) or dopamine-depleting medications (tetrabenazine and deutetrabenazine) at the time of botulinum neurotoxin treatment. This subgroup included 5 of the 32 patients who improved, 1 of the 18 who reported no change, and none of the 3 who worsened. Notably, comorbid obsessive-compulsive disorder was not predictive of treatment benefit (*P* = .74, odds ratio 1.2). Taken together, these findings support the safety and potential efficacy of botulinum neurotoxin as a treatment option for pediatric patients with motor tics. A detailed summary of patient characteristics, treatment, and outcomes can be found in the supplement section of this article (Appendix Table A).

## Discussion

This is the largest report of pediatric patients with tics treated with botulinum neurotoxin injections. A double-blind, placebo-controlled, crossover randomized controlled trial—the only one identified in the 2018 Cochrane systematic review by Pandey et al^38^—evaluated the efficacy of botulinum neurotoxin for motor tics in 18 patients (13 male), aged 15 to 55 years, each with at least 1 bothersome simple motor tic involving the face, neck, or shoulder. Participants had idiopathic tic disorders, and the specific tic selected for treatment was patient-determined. The primary outcome was the number of tics of concern per minute, measured via video analysis. Botulinum neurotoxin injections were shown to reduce both the frequency of the treated tic and the premonitory urge associated with it. However, the review emphasized the study's significant limitations, including a small sample size, relatively mild baseline tic severity, and restricted generalizability, leading to a rating of very low-quality evidence for the efficacy outcomes. Additionally, 9 participants experienced localized muscle weakness, a side effect that may have compromised the study's blinding, and no data were available regarding potential long-term risks such as immunoresistance to botulinum neurotoxin. These limitations underscore the need for further controlled studies and real-world data, particularly in pediatric populations and in those with more severe or complex tic presentations.^36,38^

Within our cohort of 50 patients, tic complexity was not found to be predictive of benefit, or lack of benefit, from botulinum neurotoxin. Interestingly, both simple and complex motor tics responded similarly well to botulinum neurotoxin treatment. This suggests that complex tics, which often involve more intricate and forceful movements, should not be excluded from consideration for botulinum neurotoxin injections. Given that patients with complex tics experienced significant improvements in tic severity, it is important for clinicians to recognize that the efficacy of botulinum neurotoxin is not limited to simpler tic patterns. In fact, patients with complex tics may derive similar therapeutic benefit from botulinum neurotoxin injections as those with simpler tics.

Botulinum neurotoxin injections have also been used in recent years in the urgent treatment of malignant tics.^39^ For example, a 21-year-old man with severe motor tics consisting of forceful twisting and extending movements of the neck resulted in a myelopathy at the C4-C5 intervertebral space.^40^ Injection of 200 U of botulinum neurotoxin in bilateral splenius muscles was associated with a marked reduction in the frequency and the intensity of the neck tics, preventing further injury. In another case, a 42-year-old man diagnosed with Tourette syndrome at age 8 developed violent dystonic neck and shoulder tics, which became disabling and frequent, ultimately resulting in quadriparesis.^40^ Pimozide, haloperidol, and risperidone were titrated to maximum tolerated doses without relief. Ultimately, 300 U of botulinum neurotoxin significantly improved his condition, reducing the frequency and severity of his dystonic tics and dramatically enhancing his quality of life.

Although our study was limited to a small sample of patients with malignant neck extension and flexion tics, the results observed in these patients suggest that botulinum neurotoxin may be clinically significant in this population. Of the 8 patients treated for these forceful and potentially harmful tics, 6 demonstrated notable improvements in the frequency and intensity of their neck tics following botulinum neurotoxin injections. Given the risk of permanent self-injury from forceful neck movements, including potential myelopathy or other serious complications, we advocate for expedited botulinum neurotoxin injection in patients with severe neck tics. Early intervention could prevent long-term physical damage, underscoring the importance of considering botulinum neurotoxin injections as part of a comprehensive treatment strategy for this high-risk group.

Given the natural waxing and waning course of tics, we elected not to include video recordings before and after botulinum neurotoxin injections, as isolated video segments may not accurately represent overall treatment response. Tic frequency and severity can vary substantially from day to day—and even within a single clinical encounter—raising the risk that video could capture a nonrepresentative snapshot and lead to misleading impressions about efficacy. Instead, we relied on longitudinal caregiver and patient reports, which better reflect real-world functional improvement over time.

Our study bears several limitations. By their nature, tics fluctuate in severity and frequency over time, making it difficult to distinguish between treatment effects and spontaneous improvements. Additionally, this study is monocentric, and limited by its retrospective, open-label design. Despite being the largest series of pediatric patients with tics treated with botulinum neurotoxin injections published to date, still larger series in the future will allow more conclusions to be drawn. A large randomized controlled trial would reduce bias and allow for the demonstration of generalizable results.

## Conclusion

This study represents the largest retrospective cohort to date describing pediatric patients with tic disorders treated with botulinum neurotoxin injections. Although most patients in our cohort were reported by caregivers to show improvement at 3-month follow-up, these observations must be interpreted cautiously given the retrospective, open-label design, lack of a control group, and reliance on subjective outcome measures. Additionally, the natural course of tic disorders—including spontaneous waxing and waning and typical improvement during adolescence—introduces potential confounding that cannot be fully accounted for in this study design.

Despite these limitations, our findings suggest that botulinum neurotoxin may be a useful therapeutic option for select pediatric patients with focal motor tics that are persistent, bothersome, or associated with risk of injury. Repeated treatment was well tolerated and commonly pursued by families who perceived benefit. Treatment decisions were individualized and informed by the distribution and severity of tics, and Botulinum neurotoxin was dosed similarly to protocols for dystonia, with gradual titration unless urgent symptom control was necessary. The lack of significant differences in response based on tic phenomenology or co-occurring neurodevelopmental conditions suggests that botulinum neurotoxin may be broadly applicable in the treatment of refractory motor tics. Rather than limiting use to specific subtypes, botulinum neurotoxin could be considered across a range of clinical presentations in children with persistent, impairing tics. Future prospective, controlled studies are needed to better define the safety, efficacy, and optimal use of botulinum neurotoxin in this population.

## Abbreviations

The following abbreviations are used in this manuscript:

BoNT: Botulinum neurotoxin
TS: Tourette syndrome
TSND: Tic disorder secondary to another neurologic diagnosis
CMTD: Chronic motor tic disorder
ADHD: Attention deficit hyperactivity disorder
OCD: Obsessive compulsive disorder
DBS: Deep brain stimulation
IQR: Interquartile range

## Appendix

**Table A.** Summary of Patient Characteristics, Treatment, and Outcomes.

Patient	Diagnosis	Age at Initial Injection	Sex	Phenomenology (Clonic/Dystonic)	Urge (Yes/No)	Comorbidities	Injection Site and Dose (U)	Outcome	Adverse Effects
**1**	TS	14	Female	Dystonic	No	ADHD, OCD	75 to each SC, 10 to each UE, 10 to each LE, 15 to each Cor.	Improved	None
**2**	TS	8	Male	Dystonic	No	ADHD	60 to each SCM, 50 to each Sub., 65 to each SC, 75 to each Sca.	Improved	None
**3**	CMTD	16	Female	Dystonic	Yes	None	65 to each SC, 40 to left Sca., 30 to right SCM	Improved	None
**4**	TSND	15	Male	Dystonic	No	ADHD, ID	10 to each Cor., 85 to each SC, 60 to Sub., 10 to each Fro., 50 to right SCM	Improved	None
**5**	TS	11	Male	Dystonic	No	ADHD	75 to each SC	Improved	None
**6**	TS	10	Male	Clonic	No	ADHD	100 to each SC, 100 to each Glu. 150 to right Lum., 100 to left Lum.	Improved	None
**7**	TS	12	Female	Clonic	Yes	ADHD	25 to each SC, 50 to right SC, 100 to right Trap. and 80 to left Trap.	Improved	None
**8**	CMTD	18	Female	Clonic	Yes	OCD	65 to right SC, 60 to left SCM, 40 to left Sca.	Improved	None
**9**	TS	16	Female	Dystonic	Yes	ADHD	30 to each Sca., 30 to each SCM, 75 to each Trap., 125 to each Lum.	Improved	None
**10**	CMTD	14	Male	Clonic	Yes	ADHD, OCD	40 to each Sup.	Improved	Local soreness
**11**	CMTD	13	Male	Dystonic	Yes	ADHD, OCD	75 to each SC	Improved	None
**12**	TSND	18	Male	Dystonic	No	ID	65 to each SC, 50 to left SCM, 50 to Sub.	Improved	None
**13**	TS	13	Male	Clonic	Yes	ADHD	60 to each SCM, 50 to Sub.	Improved	None
**14**	TS	16	Male	Dystonic	Yes	ADHD	25 to Sub.	Improved	None
**15**	TS	14	Male	Clonic	Yes	ADHD, OCD	100 to each Trap., 50 each SC	Improved	Local discomfort
**16**	TS	13	Male	Clonic	Yes	ADHD	70 to each SC	Improved	none
**17**	TS	12	Male	Clonic	No	ADHD	65 to each SC	Improved	none
**18**	TS	13	Male	Clonic	Yes	ADHD	65 to each SC	Improved	None
**19**	TSND	18	Male	Dystonic	Yes	Autism	65 to each SC, 35 to each Sca.	Improved	None
**20**	TSND	17	Female	Clonic	No	ID	50 to right SCM, 120 to right Sca., 50 to left Sca.	Improved	None
**21**	CMTD	17	Male	Clonic	Yes	ADHD, OCD	50 to right FCR, 50 to right FCU	Improved	None
**22**	TSND	11	Female	Clonic	No	ADHD, ID, autism, OCD	65 to each SC	Improved	None
**23**	TS	8	Male	Clonic	Yes	ADHD, OCD	65 to each SC, 50 to Sub.	Improved	None
**24**	TSND	18	Male	Clonic	Yes	ID, autism	125 to each Trap., 75 to each SC, 50 to each Sca., 50 to right Delt.	Improved	None
**25**	TS	12	Male	Dystonic	Yes	ADHD	70 to Sub.	Improved	None
**26**	TS	14	Female	Dystonic	Yes	ADHD, OCD	60 to left SCM, 70 to right SC	Improved	Local weakness
**27**	CMTD	15	Male	Dystonic	Yes	ADHD, OCD	75 to each SC, 50 to right Sca.	Improved	None
**28**	TS	11	Male	Clonic	Yes	OCD	50 to left SCM, 40 to right Sca., 75 to right SC	Improved	None
**29**	CMTD	10	Female	Clonic	No	OCD	65 to each SC	Improved	None
**30**	TS	8	Male	Clonic	No	ADHD, OCD	260 to each Tri.	Improved	None
**31**	TSND	17	Male	Clonic	No	ADHD, ID, autism	70 to each SCM, 60 to Sub.	Improved	None
**32**	TSND	15	Female	Clonic	Yes	ADHD, autism, OCD	40 to each SCM	Improved	None
**33**	TS	15	Male	Clonic	Yes	ADHD	65 to each SC	No benefit	None
**34**	TS	15	Male	Dystonic	Yes	ADHD	65 to each SC, 50 to each SCM, 50 to Sub.	No benefit	Local weakness, dysphagia
**35**	TS	10	Male	Dystonic	Yes	OCD	80 to left EHL, 60 to left FHLaB	No benefit	None
**36**	TSND	10	Male	Clonic	Yes	ID	50 to each SCM, 50 to Sub., 40 to right Sca.,100 to right Trap.	No benefit	None
**37**	CMTD	10	Male	Clonic	Yes	OCD	65 to each SC, 50 to each SCM, 25 to Sub., 30 to left Sca.	No benefit	None
**38**	TS	16	Male	Dystonic	No	None	12 to each Cor., 24 to each UE, 12 to each LE, 5 to each Can., 8 to each Nas.	No benefit	None
**39**	TSND	11	Male	Dystonic	No	ADHD, autism, OCD	30 to each SCM, 50 to each SC	No benefit	None
**40**	TS	14	Male	Dystonic	Yes	ADHD, OCD	10 to each Pro., 20 to each UE, 20 to each LE. 50 to each SCM, 50 to each SC, 150 to left Tho.	No benefit	None
**41**	TS	10	Male	Dystonic	Yes	OCD	70 to each SC, 60 to each SCM, 40 to left Sca.	No benefit	None
**42**	TSND	10	Male	Dystonic	No	ID	60 to each SC	No benefit	None
**43**	TSND	10	Male	Clonic	No	ID	60 to each SC, 30 to each SCM, 30 to Sub.	No benefit	None
**44**	TS	7	Male	Clonic	Yes	ADHD, OCD	65 to each SC	No benefit	None
**45**	TS	11	Female	Dystonic	Yes	ADHD	65 to each SC	No benefit	Local soreness
**46**	TS	15	Male	Dystonic	Yes	ADHD	45 to each Mas.	No benefit	None
**47**	TS	18	Male	Dystonic	Yes	None	50 to each SCM, 70 each SC	No benefit	None
**48**	TS	11	Male	Clonic	No	ADHD	50 to each Sca., 30 to each SCM	Worsened	None
**49**	CMTD	14	Male	Dystonic	Yes	None	50 to each SC, 50 to each Sca., 50 to each Trap.	Worsened	None
**50**	TSND	15	Male	Clonic	No	ADHD, ID	40 to each Sca.	Worsened	None

Abbreviations: ADHD, Attention-deficit hyperactivity disorder; Can., lateral canthus; CMTD, chronic motor tic disorder; Cor., corrugator; EHL, extensor hallucis longus; FCR, flexor carpi radialis; FCU, flexor carpi ulnaris; FHLaB, flexor hallucis longus and brevis; Fro., frontalis; Glu., gluteus medius and gluteus minimus; ID, intellectual disability; LE, lower eyelid; Lum., lumbar paraspinals; Mas., masseter; Nas., nasalis; OCD, obsessive-compulsive disorder; Pro., procerus; SC, splenius capitis; Sca., scalene; SCM, sternocleidomastoid; Sub., submental; Sup., supinator; Tho., thoracic paraspinals; Trap., trapezius; Tri., triceps; TS, Tourette syndrome; TSND, tic disorder in the setting of another neurologic diagnosis; UE, upper eyelid.
